# STAG2: Computational Analysis of Missense Variants Involved in Disease

**DOI:** 10.3390/ijms25021280

**Published:** 2024-01-20

**Authors:** David Ros-Pardo, Paulino Gómez-Puertas, Íñigo Marcos-Alcalde

**Affiliations:** Centro de Biología Molecular Severo Ochoa, Consejo Superior de Investigaciones Científicas-Universidad Autónoma de Madrid (CSIC-UAM), C/Nicolás Cabrera, 1, 28049 Madrid, Spain; davidrp@cbm.csic.es (D.R.-P.); imarcos@cbm.csic.es (Í.M.-A.)

**Keywords:** STAG2, NIPBL, RAD21, molecular modeling, Cornelia de Lange syndrome, variant rationalization

## Abstract

The human STAG2 protein is an essential component of the cohesin complex involved in cellular processes of gene expression, DNA repair, and genomic integrity. Somatic mutations in the STAG2 sequence have been associated with various types of cancer, while congenital variants have been linked to developmental disorders such as Mullegama–Klein–Martinez syndrome, X-linked holoprosencephaly-13, and Cornelia de Lange syndrome. In the cohesin complex, the direct interaction of STAG2 with DNA and with NIPBL, RAD21, and CTCF proteins has been described. The function of STAG2 within the complex is still unknown, but it is related to its DNA binding capacity and is modulated by its binding to the other three proteins. Every missense variant described for STAG2 is located in regions involved in one of these interactions. In the present work, we model the structure of 12 missense variants described for STAG2, as well as two other variants of NIPBl and two of RAD21 located at STAG2 interaction zone, and then analyze their behavior through molecular dynamic simulations, comparing them with the same simulation of the wild-type protein. This will allow the effects of variants to be rationalized at the atomic level and provide clues as to how STAG2 functions in the cohesin complex.

## 1. Introduction

Human cohesin complex is a multimeric protein complex involved in key cellular processes such as the regulation of gene expression, DNA repair, and maintenance of 3D structure and genomic integrity. Its major components are the proteins SMC1, SMC3, RAD21 (also known as SCC1), and STAG1/2 (also known as SA-1/2), as well as other associated proteins including NIPBL, PDS5, and WAPL [1,2,3,4,5,6].

Somatic mutations in coding sequences and regulatory elements of the cohesin genes have been associated with several types of cancer. These include glioblastoma, Ewing’s sarcoma, urothelial carcinoma, and myeloid neoplasms. Among the cohesin genes, STAG2 is the most frequently mutated in cancer [7]. Congenital variants in STAG2 have also been described in association with developmental disorders, most notably Mullegama–Klein–Martinez syndrome [8,9], X-linked holoprosencephaly-13 [10], and Cornelia de Lange syndrome [11].

From a structural point of view, X-ray crystallography and cryomicroscopy studies have described the interaction of the STAG1/2 protein surface with RAD21, NIPBL, and DNA, as well as with a segment of the amino-terminal end of the transcriptional regulator CTCF, within a macromolecular complex that also includes SMC1A and SMC3 [12,13].

Although the role of STAG2 as a DNA-binding subunit is well established [14,15], its specific function within the cohesin complex is still unknown. Disease-causing mutations usually exert subtle structural effects that impair the protein function while still allowing some degree of viability. Hence, mechanistic understanding by experimental means is often challenging. In the past, we have successfully used molecular dynamic (MD) simulations to functionally rationalize variants associated with Cornelia de Lange syndrome in the context of the ATP hydrolysis mechanism at the head of the cohesin complex [16]. In this work, using information from missense mutations that affect the normal function of the protein, we rationalize the effect of such mutations on the interaction of STAG2 with each of the molecules it physically contacts and for which structural information is available: NIPBL, DNA, RAD21, and CTCF. The study of the effect of these mutations on the local environment will help to understand, on the one hand, the possible origin of the disease and, on the other, which of the specific functions are affected, helping to unveil which of these interactions are key for the function of STAG2.

## 2. Results

**Figure 1:**
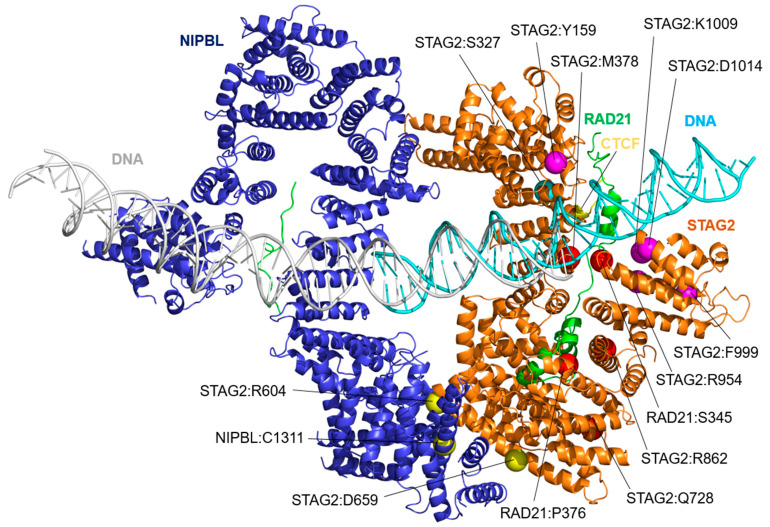
Three-dimensional model showing the relative position of STAG2 (orange) interacting with NIPBL (blue), RAD21 (green), a segment of the N-terminal domain of CTCF (yellow), and a fragment of a DNA molecule (cyan). The model is based on the Protein Data Bank structures 6WG3 [13], 4PJW [17], 6H8Q [14], and 6QNX [12]. The DNA molecule in gray corresponds to the one included in the 6WG3 structure in contact with the NIPBL molecule. Although this interaction was not analyzed in the present work, it has been included in the figure to illustrate its position. The amino acid positions of STAG2, NIPBL, and RAD21, the variants of which are analyzed, are indicated by solid spheres. The interface group they have been assigned to is indicated by the sphere color: STAG2/NIPBL interaction (yellow spheres): STAG2:Arg604Gln (R604), STAG2:Asp659His (D659), NIPBL:Cys1311Arg (C1311), and NIPBL:Cys1311His (C1311); STAG2/DNA interaction (magenta spheres): STAG2:Tyr159Cys (Y159), STAG2:Tyr159His (Y159), STAG2:Arg954Cys (R954), STAG2:Phe999Cys (F999), STAG2:Lys1009Asn (K1009), and STAG2:Asp1014Asn (D1014); STAG2/RAD21 interaction (red spheres): STAG2:Met378Val (M378), STAG2:Gln728His (Q728), STAG2:Arg862Gly (R862), RAD21:Ser345Pro (S345), and RAD21:Pro376Arg (P376); and STAG2/CTCF interaction (cyan sphere): STAG2:Ser327Asn (S327).

**Figure 2:**
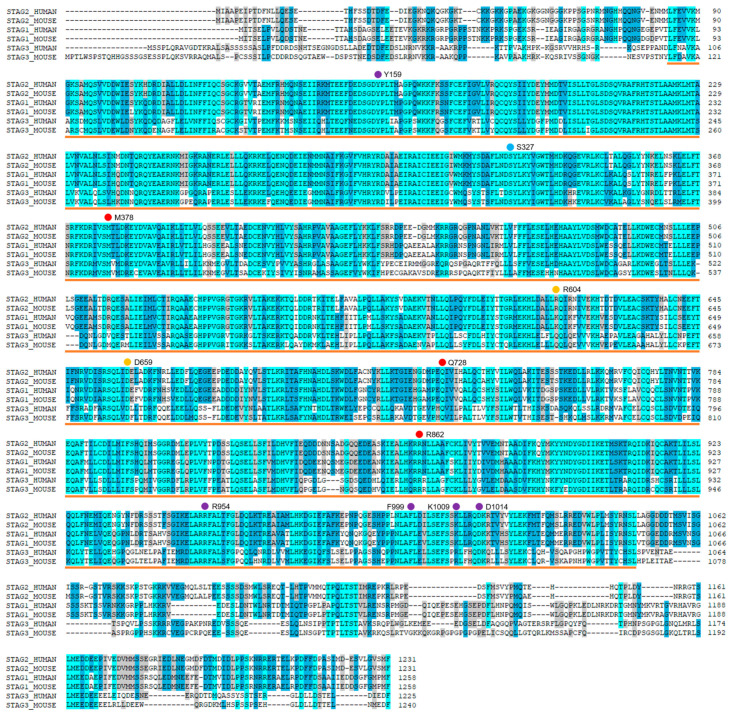
Multiple sequence alignment of mouse and human STAG1, STAG2, and STAG3 proteins (Uniprot entries: Q8WVM7, Q8N3U4, Q9UJ98, Q9D3E6, O35638, and O70576, respectively). The dots indicate the position of the human STAG2 amino acids that were analyzed, colored according to the interface to which each is associated: STAG2/NIPBL interaction (yellow dots): STAG2:Arg604Gln (R604) and STAG2:Asp659His (D659); STAG2/DNA interaction (magenta dots): STAG2:Tyr159Cys (Y159), STAG2:Tyr159His (Y159), STAG2:Arg954Cys (R954), STAG2:Phe999Cys (F999), STAG2:Lys1009Asn (K1009), and STAG2:Asp1014Asn (D1014); STAG2/RAD21 interaction (red dots): STAG2:Met378Val (M378), STAG2:Gln728His (Q728), and STAG2:Arg862Gly (R862); and STAG2/CTCF interaction (cyan dots): STAG2:Ser327Asn (S327). Sequences are colored according to their evolutionary conservation. The STAG2 sequence segment corresponding to the 3D structure is marked with an orange line.

The structure of STAG2 and the models of its interaction with RAD21, DNA, and CCTF (Figure 1) were derived from experimental structures 4PJW [17], which contains the crystallized structure of human STAG2 in contact with the human RAD21 structure; 6WG3 [13], which contains the cryo-EM structure of human NIPBL in contact with human RAD21; and 6QNX [12], with the crystallized structure of human STAG2 and RAD21 proteins in contact with a segment of human CCTF. The relative position of the STAG2, RAD21, CCTF, and DNA moieties was inferred through modeling techniques based on the structural information available. The relative positions of NIPBL and STAG2 were located using the 6WG3 structure [13] as a template, which determines the relative position of NIPBL and STAG1. Due to the high similarity between the sequence corresponding to the experimental structures of human STAG1 and STAG2, with an identity percentage of 79.7% and a similarity percentage of 90.4% (Figure 2, orange line), STAG1 scaffold could be used as a template to reliably superimpose STAG2. Finally, to determine the relative position of the DNA molecule in contact with STAG2 and RAD21, the structure 6H8Q [14], which contains the structural homologs of STAG2 and RAD21 in yeast (Scc3 and Scc1, respectively) co-crystallized with a DNA segment, was used as reference, as this is the only available structure of a STAG2 homolog in contact with DNA.

In the present work, using MD techniques, we have simulated STAG2 in a complex with the macromolecules for which structural information on their physical interaction is available: NIPBL, DNA, RAD21, and a segment of the N-terminal end of CTCF. These simulations were performed for wild-type proteins, as well as for a total of 16 missense mutations (Table 1) that were located at the interface between STAG2 and the other complex components: 12 STAG2, 2 NIPBL, and 2 RAD21 variants. Figure 1 shows a model of the interaction of STAG2 with the different molecules in its environment: NIPBL, DNA, RAD21, and CTCF, as well as the position of the amino acids, the variants of which will be studied. All STAG2 variants are located in highly conserved segments (Figure 2).

The variants have been divided into four groups according to the interface they are closest to (Figure 1): STAG2/NIPBL interaction, STAG2/DNA interaction, STAG2/RAD21 interaction, and STAG2/CTCF interaction. Each of the variants, as well as the corresponding controls, have been subjected to a 200 ns long molecular dynamics simulation, the stability of which has been assessed by measuring the Root Mean Square Deviation (RMSD, Supplementary Figures S1–S3).

### 2.1. STAG2/NIPBL Interaction

**Figure 3:**
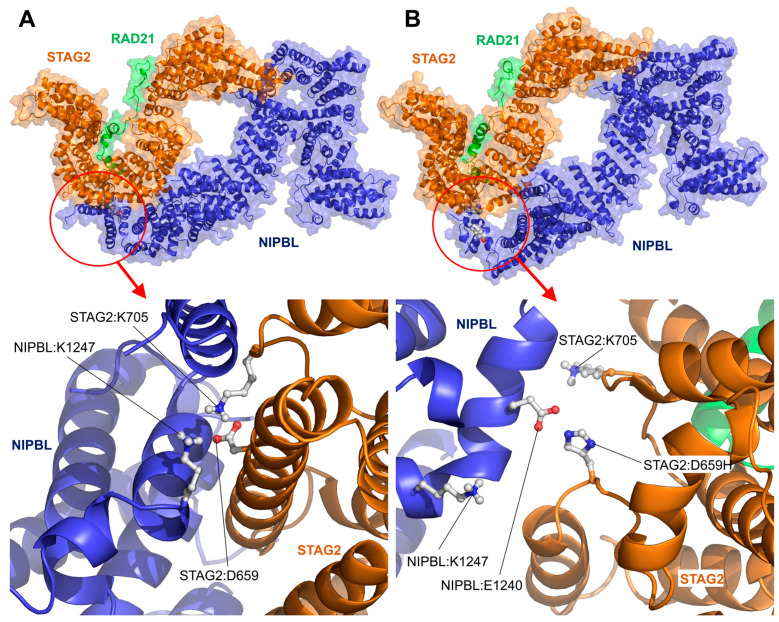
STAG2/NIPBL interaction: STAG2:Asp659His variant. Structure of the interaction interface between NIPBL and STAG2 in presence of the wild-type STAG2:Asp659 residue (**A**) and variant STAG2:Asp659His (**B**) after a 200 ns long MD simulation. (**Upper panels**) Structure of the STAG2-NIPBL complex after MD simulation. (**Bottom panels**) Zoom on the area where the STAG2:Asp659 residue is located on the interaction surface between the two proteins. (**A**) The negatively charged residue STAG2:Asp659 (D659) forms a stable interaction with the positively charged amino acids STAG2:Lys705 (K705) and NIPBL:Lys1247 (K1247). (**B**) The presence of a histidine at the position corresponding to the Asp659 residue in the STAG2:Asp659His (D659H) variant causes the separation of the positive amino acids STAG2:Lys705 (K705) and NIPBL:Lys1247 (K1247) from their original position. Instead, STAG2:Asp659His binds to the residue NIPBL:Glu1240 (E1240). This structural change causes the noticeable disorganization of the neighboring structures and replaces two salt-bridges with a single polar interaction, which can be expected to significantly weaken the interaction between NIPBL and STAG2.

Four variants are included in this group, two in the STAG2 sequence (STAG2:Arg604Gln and STAG2:Asp659His), and two others in the NIPBL sequence (NIPBL:Cys1311Arg and NIPBL:Cys1311Tyr), all four of which are located at the interface between the two proteins. In the case of the STAG2:Asp659His variant, Figure 3 shows an image of the local structure after 200 nanoseconds (ns) of productive MD simulation of both the wild-type protein (STAG2:Asp659) and the STAG2:Asp659His variant. In the wild-type protein, the negatively charged amino acid STAG2:Asp659 was observed to interact stably with STAG2:Lys705 and, somewhat less stably throughout the simulation, with NIPBL:Lys1247. This interaction contributes to the correct localization of the STAG2 and NIPBL surfaces in contact with each other (Figure 3A). In the case of the STAG2:Asp659His variant, the substitution of Asp659 by a positively charged amino acid, such as His, causes a number of structural changes (Figure 3B; Supplementary Figure S3). Due to their positive charge, NIPBL:Lys1247 and STAG2:Lys705 no longer interact with STAG2:Asp659His and move to the surrounding area. This fact allows an amino acid that was previously far away from this zone, NIPBL:Glu1240, to change its position and come into contact with STAG2:Asp659His. This maintains the local interaction between the surfaces of both proteins but at the cost of a disorganization of the alpha-helix containing STAG2:Asp659His, which is split into two smaller alpha-helices, and a change in the local relative position of NIPBL with respect to STAG2. Furthermore, the replacement of two salt bridges by a single polar interaction can be expected to significantly weaken the interaction between both proteins. The average distance between the amino acids in the NIPBL helix containing the NIPBL:Lys1247 residue and the STAG2 helix containing the STAG2:Asp659His variant changes from a value of 11.06 ± 1.24 Angstrom (Å) for the wild-type protein over the 200 ns MD trajectory to a value of 27.71 ± 1.32 Å in the last 50 ns of the MD trajectory for the STAG2:Asp659His variant. Therefore, the described effect may explain, on the one hand, the deleterious effect of the variant by the noticeable interface disruption, but, on the other, may also explain the partial retention of its function due to a new contact between STAG2:Asp659His and NIPBL:Glu1240.

In the STAG2:Arg604Gln variant, no significant changes in the local environment were observed during the MD simulation, with respect to the wild-type condition (Supplementary Figures S1 and S2). STAG2:Arg604 does not seem to be involved in the formation of salt bridges with charged amino acids of NIPBL, so the exchange of STAG2:Arg604 for Gln (a polar but uncharged amino acid) does not seem to promote significant changes. Both STAG2:Arg604 and STAG2:Arg604Gln appear to interact with the same amino acids and in a similar geometry. The deleterious effect might be caused by the non-ionic character of glutamine in comparison with aspartic acid, resulting in weaker interactions with the neighboring polar residues, by possible problems during protein folding, by effects that involve other proteins not described in the model, or by any other effect beyond the scope of this work.

**Figure 4:**
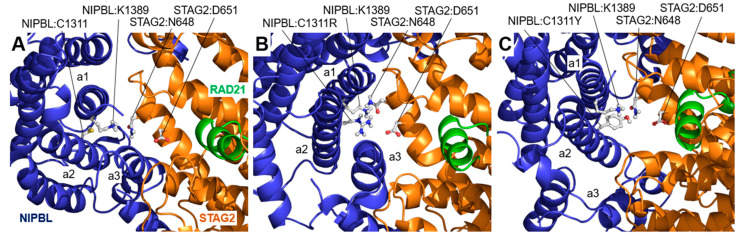
STAG2/NIPBL interaction: NIPBL:Cys1311Arg and NIPBL:Cys1311Tyr variants. Interface between wild type NIPBL and STAG2 in the vicinity of the amino acid NIPBL:Cys1311 (C1311) after a 200 ns long MD simulation. (**A**) In the wild-type interface, close to the NIPBL:Cys1311 residue (located in an alpha-helix in the HEAT repeat structure, marked (**a2**)), the amino acid NIPBL:Lys1389 (K1389) (located in the adjacent helix, marked (**a1**)) interacts with the amino acid STAG2:Asn648 (N648), contributing to the stability of the local structure of the interface between NIPBL and STAG2. (**B**) In the NIPBL:Cys1311Arg (C1311R) variant, the amino acid NIPBL:Arg1311 interacts with the amino acids STAG2:Asn648 (N648) and STAG2:Asp651 (D651), which causes the displacement of the (**a1**)- and (**a2**)-labeled helices of NIPBL during the MD simulation, locally disorganizing the interface structure. (**C**) A similar effect is observed for the NIPBL:Cys1311Tyr (C1311Y) variant, where the structural change is enhanced by the large displacement of a third helix of the HEAT repeat structure of NIPBL, labeled (**a3**).

Two variants have been described in the NIPBL:Cys1311 position: NIPBL:Cys1311Arg and NIPBL:Cys1311Tyr. In the wild-type protein, the amino acid NIPBL:Cys1311 is localized in one of the HEAT repeat structures of NIPBL that is close to but not in contact with STAG2 (Figure 4A). Near NIPBL:Cys1311, the residue NIPBL:Lys1389 is located in direct contact with STAG2:Asn648 on the surface of STAG2, contributing to the stability of the interaction between the two proteins. In the NIPBL:Cys1311Arg variant (Figure 4B), after MD simulation, the amino acid NIPBL:Lys1389 is slightly shifted due to the positioning of the mutated amino acid NIPBL:Cys1311Arg in its place, which also interacts with the STAG2:Asn648 residue. The new localization of NIPBL:Cys1311Arg facilitates the approach of an amino acid that was not previously in the environment: the negatively charged STAG2:Asp651. Thus, the variant seems to exacerbate the interaction between NIPBL and STAG2 due to the electrostatic attraction between the face of the alpha helices containing the positively charged amino acids NIPBL:Lys1389 and NIPBL:Cys1311Arg and the negatively charged amino acid STAG2:Asp651. This change is indeed accompanied by a shift of the alpha-helices containing NIPBL:Lys1389 and NIPBL:Cys1311Arg towards the position occupied by STAG2:Asp651. In the case of the NIPBL:Cys1311Tyr variant (Figure 4C), although the interaction between NIPBL:Cys1311Tyr (polar but uncharged) and STAG2:Asp651 is not established, a large displacement is observed, not only of the helices containing the amino acids NIPBL:Lys1389 and NIPBL:Cys1311Tyr, but also of a third NIPBL alpha-helix (named a3 in Figure 4C), which shifts out of the local environment during the simulation, causing a large structural change at the contact surface between the two proteins. The average distance between the amino acids in helices a1 and a2, containing residues NIPBL:Cys1311 and NIPBL:Lys1389, the STAG2 helix, and the amino acid STAG2:Asp651, varies from a mean of 18.18 ± 0. 62 Å over the 200 ns MD trajectory of the wild-type protein to mean values of 12.23 ± 1.83 Å and 13.85 ± 1.44 Å over the last 50 ns of the MD trajectory for the NIPBL:Cys1311Arg and NIPBL:Cys1311Tyr variants, respectively. The structural changes induced by the STAG2:Asp659His, NIPBL:Cys1311Arg, and NIPBL:Cys1311Tyr variants are associated with high RMSD values measured in the corresponding regions (Supplementary Figure S3).

### 2.2. STAG2/DNA Interaction

**Figure 5:**
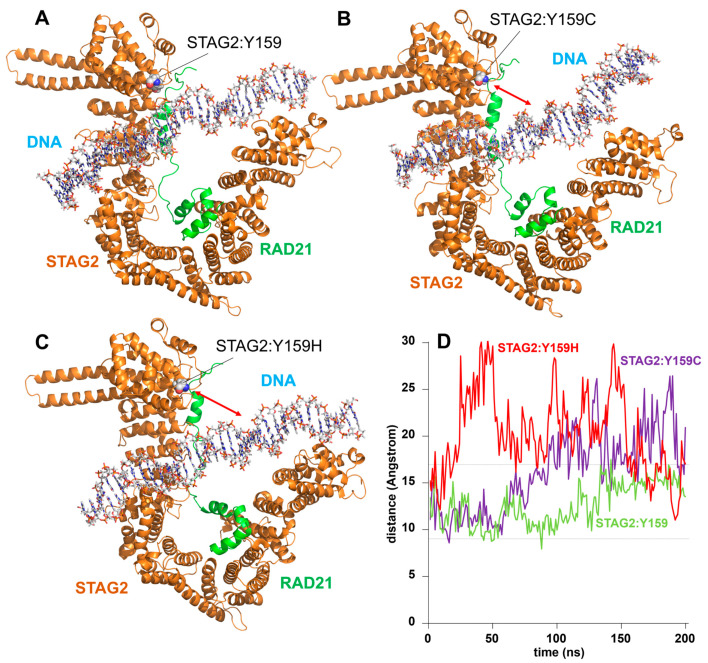
STAG2/DNA interaction: STAG2:Tyr159Cys and STAG2:Tyr159His variants. Model of the interaction of the STAG2-RAD21 dimer with a DNA molecule after 200 ns of MD simulation. (**A**) In the wild-type dimer, the position of the STAG2:Tyr159 (Y159) amino acid in a domain of STAG2 contacting the DNA molecule is indicated. (**B**) Model of the interaction of STAG2-RAD21 dimer with DNA in the case of the STAG2:Tyr159Cys (Y159C) variant. The red arrow indicates the separation between the DNA molecule and the STAG2 domain. (**C**) Model of the interaction of STAG2-RAD21 dimer with DNA in the case of the STAG2:Tyr159His (Y159H) variant. (**D**) Plot showing the variation of the distance from the alpha carbon of STAG2:Tyr159 (Y159, green line), STAG2:Tyr159Cys (Y159C, purple line), and STAG2:Tyr159His (Y159H, red line) to the nearest DNA phosphate group over 200 ns of unrestricted MD simulation. The distance in the case of the wild-type molecule ranges between 9 and 17 Å (dashed lines), whereas, in the case of both variants, the oscillation is much larger, reaching separation values of up to 30 Å.

**Figure 6:**
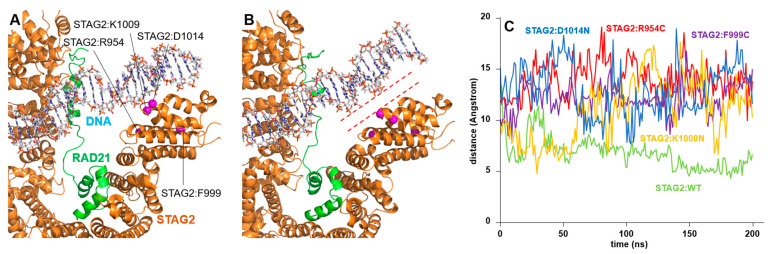
STAG2/DNA interaction: STAG2:Arg954Cys, STAG2:Phe999Cys, STAG2: Lys1009Asn, and STAG2:Asp1014Asn variants. Model of the interaction of the STAG2-RAD21 dimer with a DNA molecule after 200 ns of MD simulation. (**A**) The position of residues STAG2:Arg954 (R954), STAG2:Phe999 (F999), STAG2: Lys1009 (K1009), and STAG2:Asp1014 (D1014) located in a DNA contacting domain of wild-type STAG2-RAD21 dimer are shown. (**B**) Model of the interaction between DNA and the STAG2-RAD21 dimer in the case of the STAG2:Arg954Cys (R954C) variant during MD simulation. The dashed lines indicate the separation between the DNA strand and the STAG2 domain where the variants are located. (**C**) Plot showing the variation of the distance between the alpha carbon of a residue in the contact surface of STAG2 and the nearest DNA phosphate group over 200 ns of unrestricted MD simulation in the following conditions: wild-type STAG2 (green line), STAG2:Arg954Cys (R954C, red line), STAG2:Phe999Cys (F999C, purple line), STAG2: Lys1009Asn (K1009N, yellow line), and STAG2:Asp1014Asn (D1014N, blue line). The distance in the case of the wild-type molecule stabilizes at a position close to 5 Å; the analysis of the four variants shows that the distance oscillates strongly and reaches values of almost 20 Å at certain times.

Six STAG2 variants are included in this group. Two of them correspond to a mutation in the same residue: STAG2:Tyr159Cys and STAG2:Tyr159His (Figure 5), while the other four are grouped on the other side of the DNA: STAG2:Arg954Cys, STAG2:Phe999Cys, STAG2:Lys1009Asn, and STAG2:Asp1014Asn (Figure 6). The amino acid STAG2:Tyr159 localizes to a region of STAG2 in contact with DNA, although the amino acid itself has no direct interaction with the nucleotide chain (Figure 5A). In the STAG2:Tyr159Cys and STAG2:Tyr159His variants, the amino acid changes are not particularly drastic because both the original amino acid (Tyr) and the variants (Cys and His) are all polar residues. However, the small changes associated with these substitutions seem to correspond to a greater flexibility in the local interaction between the protein moiety located close to the mutated amino acid and the DNA molecule. Figure 5B,C show an example of one of the separation moments between the DNA and the STAG2 domain during the simulation corresponding to the STAG2:Tyr159Cys and STAG2:Tyr159His variants, respectively. Figure 5D shows the variation, along the MD trajectory, of the distance between the alpha carbon of the STAG2:Tyr159 amino acid (or of the STAG2:Tyr159Cys and STAG2:Tyr159His variants) and the nearest phosphate group of the DNA chain at the beginning of the simulation. The graph shows how this distance remains within the range of 9 to 17 Angstrom for the case of the wild-type amino acid. On the other hand, in the two mutants, a much larger oscillation is observed, reaching values of up to 30 Å at some moments of the simulations (mean values of 19.69 ± 3.05 and 17.00 ± 3.44 Å for STAG2:Tyr159Cys and STAG2:Tyr159His variants, respectively, in the last 50 ns of the trajectory).

The four variants STAG2:Arg954Cys, STAG2:Phe999Cys, STAG2:Lys1009Asn, and STAG2:Asp1014Asn are located in region of STAG2 that is in close contact with the DNA double strand (Figure 6A). As in the case of the STAG2:Tyr159Cys and STAG2:Tyr159His mutants, no significant structural change in the surrounding domain is observed in these four variants. In the case of the STAG2:Arg954Cys, STAG2:Lys1009Asn, and STAG2:Asp1014Asn variants, the subtlety of the effect may be due to the resulting amino acid substitutions retaining the polar character (Cys, Asn, and Asn). Nevertheless, none of these substitutions retain the ionic nature of the wild-type amino acids, which might significantly weaken the interaction with the DNA. On the other hand, STAG2:Phe999 is buried in the domain structure within a hydrophobic cluster, yet the switch to a polar amino acid (Cys) does not seem to cause a large local change in the 3D structure of the backbone, probably because of the smaller size of STAG2:Phe999Cys; however, some effects during protein folding can be expected given the change from a non-polar to polar amino acid in the context of a hydrophobic pocket.

In contrast, STAG2:Arg954Cys, STAG2:Phe999Cys, STAG2:Lys1009Asn, and STAG2:Asp1014Asn exhibit an increased variability in the distance between the STAG2 region and the DNA molecule (Figure 6), similarly to what was observed on STAG2:Tyr159His. In the wild-type protein, the contact between the domain and the DNA is stable, moving around values close to 5 Å towards the end of the MD trajectory. In contrast, in the case of the four mutants, this distance is much more unstable, oscillating between values of 10 and 20 Å (mean values of 13.60 ± 1.36, 12.75 ± 1.42, 11.86 ± 2.15, and 13.70 ± 1.88 Å for STAG2:Arg954Cys, STAG2:Phe999Cys, STAG2:Lys1009Asn, and STAG2:Asp1014Asn variants, respectively).

Considering that the main function described for STAG2 is specifically to bind to the DNA molecule, these variations in the interaction between the two molecules observed in the six variants studied are very likely to affect the stability and geometry of the interaction and its associated function.

### 2.3. STAG2/RAD21 Interaction

**Figure 7:**
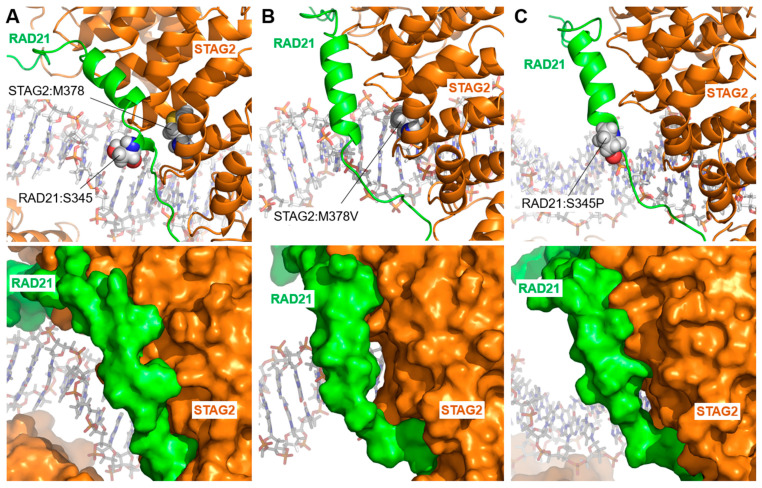
STAG2/RAD21 interaction: STAG2:Met378Val and RAD21:Ser345Pro variants. Interface between STAG2 and RAD21 after 200 ns of unconstrained MD simulation. (**A**) (**Upper panel**) position of the amino acids STAG2:Met378 (M378) and RAD21:Ser345 (S345) at the interface between wild-type STAG2 and RAD21. (**Lower panel**) the same structure as in the upper panel, illustrating the surface of the proteins, showing the close contact between the two molecules. (**B**) Structure of the STAG2-RAD21 interface for the STAG2:Met378Val (M378V) variant. A change in the structure of the adjacent RAD21 alpha-helix (**upper panel**) is observed, resulting in a separation between the surfaces of the two proteins (**lower panel**). (**C**) Structure of the STAG2-RAD21 interface in the case of the RAD21:Ser345Pro (S345P) variant. In this case, there is also a change in the structure of the RAD21 alpha-helix where the variant is localized (**upper panel**), forcing a separation at the interface of the two proteins (**lower panel**).

Three STAG2 variants (STAG2:Met378Val, STAG2:Gln728His, and STAG2:Arg862Gly) and two RAD21 variants (RAD21:Ser345Pro and RAD21:Pro376Arg) belong to this group. Two of them (STAG2:Met378Val and RAD21:Ser345Pro) localize very close to each other on the interaction surface between RAD21 and STAG2 (Figure 7A). Figure 7B shows the effect of the STAG2:Met378Val variant on the interaction between STAG2 and RAD21. Although the change from STAG2:Met378 to Val is conservative, as are both hydrophobic amino acids, the difference in size between the two is likely to induce a change in local structure that is transmitted to the neighboring RAD21 protein. In RAD21, the alpha helix contacting STAG2 at this site is displaced and undergoes a change in morphology, resulting in a separation between the two surfaces, from a mean value of 6.82 ± 0.49 Å in the wild-type trajectory to a mean distance of 10.65 ± 2.04 in the last 50 ns of the STAG2:Met378Val variant trajectory, as shown in Figure 7B (bottom). In the case of the RAD21:Ser345Pro variant, located in a position close to the residue STAG2:Met378, the non-conservative substitution of RAD21:Ser345 by Pro causes a conformational change in the RAD21 alpha-helix where the mutated amino acid is located (Figure 7C, Supplementary Figure S3). As in the case of the STAG2:Met378Val variant, the RAD21:Ser345Pro mutation results in the surface separation of the two proteins (mean distance of 9.02 ± 1.09 Å in the last 50 ns of the trajectory). In both cases, this separation leads to a change in the stability of the coupling between the two molecules, affecting both the formation of the RAD21–STAG2 dimer, which is necessary for the complex to work properly, and the potential interaction of STAG2 with the DNA molecule.

**Figure 8:**
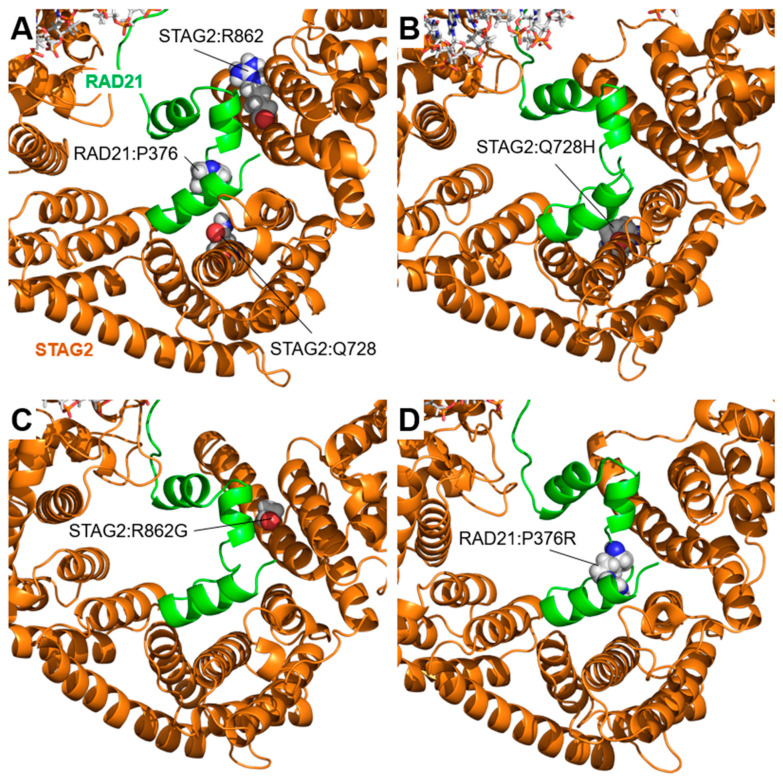
STAG2/RAD21 interaction: STAG2:Gln728His, STAG2:Arg862Gly, and RAD21:Pro376Arg variants. Model of the interaction between STAG2 and RAD21 after 200 ns of MD simulation. (**A**) The positions of the residues STAG2:Gln728 (Q728), STAG2:Arg862 (R862), and RAD21:Pro376 (P376) in the interface between wild-type STAG2 and RAD21 are indicated. (**B**–**D**). Models of the interaction between STAG2 and RAD21 corresponding to the variants STAG2:Gln728His (Q728H, panel (**B**)), STAG2:Arg862Gly (R862G, panel (**C**)), and RAD21:Pro376Arg (P376R, panel (**D**)). No major changes were observed in any of the three variants analyzed.

Figure 8A shows the position of the other three variants (STAG2:Gln728His, STAG2:Arg862Gly, and RAD21:Pro376Arg) in a position close to each other on the interaction surface between STAG2 and RAD21, although far from the area where the two variants discussed above are located. Figure 8B–D show the domain structure after the MD simulation of STAG2:Gln728His, STAG2:Arg862Gly, and RAD21:Pro376Arg, respectively. The figure shows how, although some of the changes are clearly non-conservative, no major shifts in the relative position of the two proteins are apparent. As in the case of the STAG2:Arg604Gln variant mentioned above, the effect of the mutations may have to be explained by the altered interaction with components not present in this model, possible problems during protein folding, or other effects out of the scope of the methodology applied in this work.

### 2.4. STAG2/CTCF Interaction

**Figure 9:**
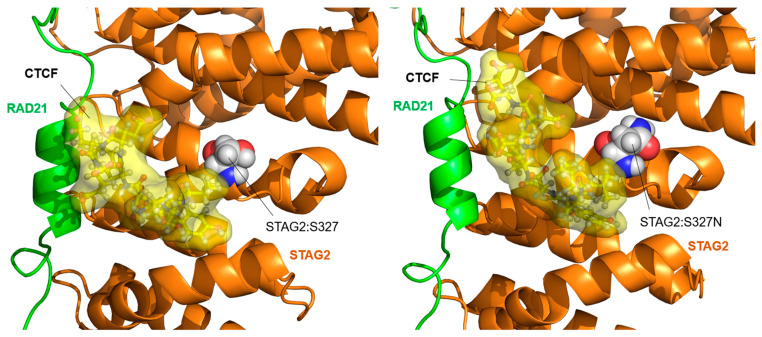
STAG2/CTCF interaction: STAG2:Ser327Asn variant. Model of the interaction between STAG2-RAD21 and a peptide located in the N-terminal domain of CTCF, after 200 ns of MD simulation. (**Left**) The position of the wild-type amino acid STAG2:Ser327 (S327) in an alpha-helix in contact with the position of the CTCF peptide is indicated. (**Right**) Model of the interaction in the case of the STAG2:Ser327Asn (S327N) variant. A slight shift in the position of the CTCF peptide can be observed, separating it from the contact with RAD21.

The interaction segment of CTCF with STAG2 consists of only nine amino acids located in the N-terminal domain of the protein [12]. Only one of the studied STAG2 variants, STAG2:Ser327Asn, is located near this binding site. Figure 9 shows the position of the STAG2-bound CTCF segment in the wild-type protein (left) and in the STAG2:Ser327Asn variant (right). Although no significant structural change is observed when replacing Ser with Asn (both are uncharged polar residues), the geometry of the CTCF segment interaction changes slightly during the MD simulation, moving away from the contact with RAD21.

## 3. Discussion

The computational simulation by the MD of the motions associated with macromolecular structures is a tool that allows us to rationalize, at the atomic level, the effects that certain variants in the sequence of a protein can have on its function and on its relationship with other molecules. For example, in a previous work of our group focusing on Cornelia de Lange syndrome, the computational modeling of ATP hydrolysis in the head domain of the SMC1A and SMC3 proteins was able to illustrate the changes associated with a pathogenic variant in SMC3 that functionally alters this mechanism [16].

To our knowledge, no previous work has been published that has studied the effect of the different STAG2 variants or the proteins that interact with it at the biochemical or biophysical level. For this reason, we believe that the use of molecular dynamics simulation techniques is one of the few ways available to approach an atomic or molecular rationalization of the phenotypic effect of each of the variants.

STAG2 is an essential protein for the structure and function of the cohesin complex, although its specific role beyond its ability to bind DNA is unknown. In the present work, we have used information from pathogenic variants of STAG2 and its interacting proteins, all in evolutionary conserved positions, to advance our understanding of the function of STAG2 and the causes of disease, using computational modeling and MD simulation techniques. The variants studied were divided into four groups according to the closest neighboring molecule in the structure: STAG2/NIPBL interaction, STAG2/DNA interaction, STAG2/RAD21 interaction, and STAG2/CTCF interaction.

Of the four variants close to the STAG2/NIPBL interaction, three of them have shown similar behavior, strongly modifying the contact structure between the two proteins. Some works have shown how the relationship of NIPBL with STAG2 is different from the relationship with STAG1 [5,27], which opens the field to study in the future, by computational modeling, the interaction of STAG1 with NIPBL, and its differences with the interaction observed with STAG2 in the current work.

In the case of the variants affecting the STAG2/DNA interaction, all of them showed a similar behavior, destabilizing the coupling between the two molecules, with large variations in the distance between the DNA and the nearby domains. Considering the major role of STAG2 in DNA binding, these observations open the way to a more detailed study of the interaction, using advanced modeling and dynamic simulation techniques. In our group, we are currently working in this direction, which will hopefully contribute to a better understanding of the role of STAG2 in the cohesin complex machinery.

Among the variants related to the STAG2/RAD21 interaction, two of them, one in RAD21 (RAD21:Ser345Pro) and another in STAG2 (STAG2:Met378Val), have shown very similar behavior, partially disrupting the interaction zone between the two proteins. The effect of RAD21:Ser345Pro described in this work is very similar to that previously described by our group, although this work did center around a simulation performed in the absence of DNA [24]. The consistency between the two observations in two different simulation conditions boosts the confidence in these results. Further studies are needed to fully understand the role that the interaction between STAG2 and RAD21 plays, not only for the co-localization of both, but also as part of their structural mechanism of action.

In the area of the STAG2/CTCF interaction, the changes observed in the simulation should be taken with caution. The small size of the CTCF peptide bound to STAG2 makes it very sensitive to small perturbations of the MD environment. It will be necessary to generate structures that include a larger region of CTCF, including its C-terminal domain, in order to advance the modeling of the interaction between the two proteins.

One of the points to be considered when studying the effect of variants in the proteins of the cohesin complex is the effect of dominance. Most of the variants found in patients are either heterozygous or, for the genes located on the X chromosome (SMC1A and HDAC8), correspond to amino acid changes with limited effect on function [8,11,18,19,20,21,22,23,24,25,26]. A reasonable hypothesis would be that, in the case of drastic mutations substantially affecting protein folding and, thus, profoundly impairing the cohesin complex function, the effect on cell division and gene regulation in embryonic stages would be so severe that it would not allow progression. In the case of the variants analyzed in this work, all but four positions (STAG2:Tyr159, STAG2:Phe999, STAG2:Met378, and NIPBL:Cys1311) correspond to surface residues exposed to the solvent and are therefore more involved in interactions with neighboring molecules than in internal interactions related to protein folding. Thus, STAG2 Arg604Gln and Asp659His variants would affect NIPBL binding; STAG2 Arg954Cys, Lys1009Asn, and Asp1014Asn variants would affect DNA interaction; STAG2 Gln728His, Arg862Gly, RAD21 Ser345Pro, and Pro376Arg variants would be involved in RAD21–STAG2 interaction; and the STAG2 Ser327Asn variant would be associated with STAG2 binding to CTCF. In addition to these interactions, their possible role in undescribed contacts with other macromolecules in the environment cannot be excluded. In the case of STAG2 variants of residues buried inside the protein (Tyr159Cys, Tyr159His, Phe999Cys, and Met378Val), the change does not seem to be related to changes in the local folding of the corresponding domains, but to local modifications transmitted to the surface amino acids close to them, which are involved in the interaction with other macromolecules (NIPBL, DNA, or RAD21). The case of the NIPBL variants Cys1311Arg and Cys1311His is somewhat mixed, as the electrostatic charge of the variants seems to push these residues from the hidden position of the original amino acid to a more surface-exposed position in contact with STAG2. In any case, as shown in Supplementary Figures S1 and S2, most of the variants have little effect on the overall structure of the protein regions. The only exceptions would be the STAG2:Asp659His, NIPBL:Cys1311Arg, NIPBL:Cys1311Arg, and RAD21:Ser345Pro variants (Supplementary Figure S3), the presence of which induces significant changes in the regions in which they are located. Nevertheless, even in these cases, the effect of these mutations is local and does not seem to profoundly disrupt the folding of the protein.

## 4. Materials and Methods

The structure of STAG2 and the models of its interaction with RAD21 and DNA were obtained by homology modeling, using the PDB entry 4PJW [17] as a template, which describes the interaction of STAG2 with RAD21. The DNA position was modeled using the 3D structure of the SCC3–SCC1 complex bound to DNA, solved by X-ray crystallography (PDB entry 6H8Q [14]), as a reference. The DNA model was constructed using x3DNA [28]. Gaps were modeled by combining predictions from Phyre 2 [29] and SwissModel [30]. The NIPBL interaction model was obtained by homology modeling from the cryo-EM structure of the human SMC1A-SMC3-RAD21-STAG1-NIPBL-DNA complex (PDB id: 6WG3 [13]). The interaction of STAG2 with CTCF was modeled from PDB ID: 6QNX [12], which contains the structure of a segment of the N-terminal domain of CTCF bound to STAG2.

The 3D coordinates of STAG2 and the models of its interaction with RAD21, DNA, and CCTF were obtained from experimental structures: 4PJW [17] (crystallized structure of human STAG2 and human RAD21); 6WG3 [13] (cryo-EM structure of human NIPBL, RAD21, SMC1A, SMC3, STAG1, and DNA); and 6QNX [12] (crystallized structure of human STAG2, RAD21, and a segment of CCTF). The relative positions of NIPBL and STAG2 were determined by directly superimposing the STAG2 structure on the position of STAG1 in the 6WG3 structure [13]. The initial position of the DNA molecule in contact with STAG2 and RAD21 was determined using, as a guide, the 6H8Q structure [14], which contains the structural homologues of STAG2 and RAD21 in yeast (Scc3 and Scc1, respectively), co-crystallized with a DNA segment.

The wild-type models were parameterized with the LEaP module of Amber Tools [31], using the Amber ff14SB force field [32] for the protein moieties and the Amber OL15 force field [33] for the DNA chain. The structures were solvated with the TIP3P solvent model [34], adding Na+ or Cl- ions to neutralize the charge on the system. 

Molecular dynamics simulations were conducted using the Amber18 software package (University of California, San Francisco, CA, USA), employing the Particle Mesh Ewald (PME) method for non-bonded interactions with a cutoff distance of 8 Å. The temperature was regulated through the Langevin thermostat, maintaining a fixed temperature of 297 K with a collision frequency of 1 ps. Hydrogen bond constraints were implemented using the SHAKE algorithm, enabling a simulation timestep of 2 fs. To uphold NPT conditions (constant number of particles, pressure, and temperature), the pressure coupling was managed by a Monte Carlo Barostat set to 297 K and 1 bar.

The initial model structures underwent 10,000 cycles of energy minimization, followed by a 1 ns restrained equilibration phase, smoothly raising the temperature to 297 K, after which restraints were gradually removed over 10 ns. Subsequently, each system was subjected to a 200 ns long free MD production phase.

Simulations of the different missense variants were performed by substituting the corresponding wild-type amino acid for the variant, following the same modeling and MD simulation steps previously described. The positions of amino acids and DNA strands throughout the MD trajectories were analyzed using cpptraj [35] and VMD [36]. Figures were generated with Pymol (Schrödinger, New York, NY, USA).

Multiple sequence alignments of human and mouse STAG1/2/3 proteins were generated using MUSCLE [37] and visualized and colored according to their evolutionary conservation using belvu [38]. 

## Figures and Tables

**Table 1:** Missense variants analyzed.

Interaction	Variant	References
STAG2/NIPBL	STAG2:Arg604Gln	[8,18]
	STAG2:Asp659His	[11]
	NIPBL:Cys1311Arg	[19]
	NIPBL:Cys1311His	ClinVar VCV001697856.1 [20]
STAG2/DNA	STAG2:Tyr159Cys	[18]
	STAG2:Tyr159His	[21]
	STAG2:Arg954Cys	[22]
	STAG2:Phe999Cys	ClinVar VCV001395977.2 [20]
	STAG2:Lys1009Asn	[9]
	STAG2:Asp1014Asn	[23]
STAG2/RAD21	STAG2:Met378Val	[11]
	STAG2:Gln728His	ClinVar VCV001299204.12 [20]
	STAG2:Arg862Gly	ClinVar VCV000521058.3 [20]
	RAD21:Ser345Pro	[24]
	RAD21:Pro376Arg	[24,25]
STAG2/CTCF	STAG2:Ser327Asn	[26]

Variants analyzed, grouped according to their proximity to each interacting molecule.

## Data Availability

The data analyzed during this study are included in this published article. Additional supporting data are available from the corresponding authors upon reasonable request.

## Supplementary Materials

The supporting information can be downloaded at: https://www.mdpi.com/article/10.3390/ijms25021280/s1.
