# Supplementary material for: STAG2: Computational Analysis of Missense Variants Involved in Disease
Source: Int J Mol Sci. 2024 Jan 20;25(2):1280. doi: 10.3390/ijms25021280 (PMC10816197; doi:10.3390/ijms25021280)
Supplement: Supplementary file 1 [file ijms-25-01280-s001.zip › ijms-2808742-supplementary.pdf]

Supplementary Material to:

STAG2: Computational analysis of missense variants involved in disease.

Authors: David Ros-Pardo, Paulino Gómez-Puertas & Íñigo Marcos-Alcalde

- Supplementary Figure S1
- Supplementary Figure S2
- Supplementary Figure S3

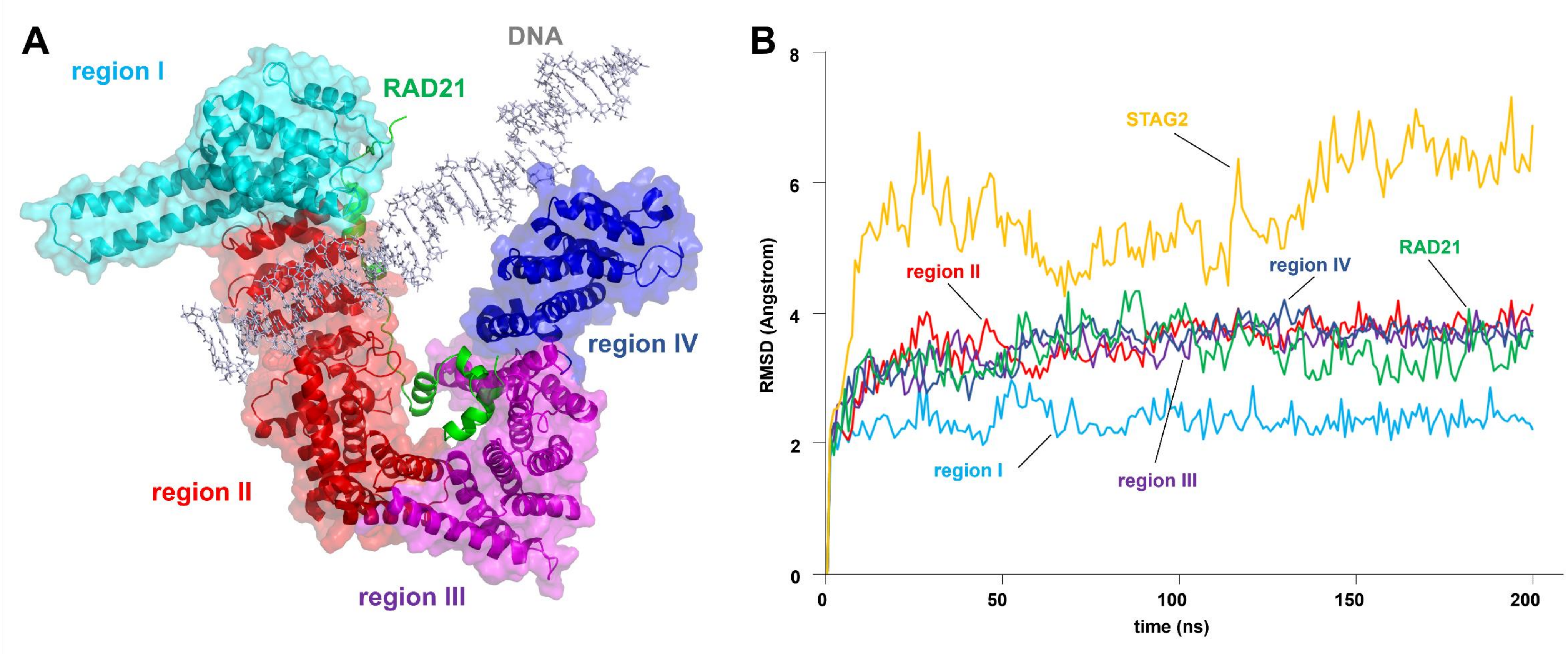

Supplementary Figure S1. Root Mean Square Deviation (RMSD) analysis of the structure of STAG2 and RAD21 during molecular dynamics simulations. A. Region partitioning of the STAG2 structure. During the molecular dynamics trajectory, due to the internal flexibility of its V-shaped structure, the entire STAG2 protein undergoes a series of oscillations that produce very high values in the RMSD measurement, masking any other variation of lesser amplitude. For this reason, the STAG2 protein has been subdivided into four regions the structure of which remains internally stable: region I (residues 84 to 302; cyan), region II (residues 303-647; red), region III (residues 648-880; purple) and region IV (residues 881-1048; blue). The segment corresponding to RAD21 is treated as an additional region (green color). B. Measured RMSD values (in Angstrom) during the 200 ns molecular dynamics simulation of the entire structure of STAG2 protein (orange line, showing large oscillations), the RAD21 segment (green line), and each of the four regions of STAG2 (I, II, III, and IV, cyan, red, purple, and blue lines, respectively).

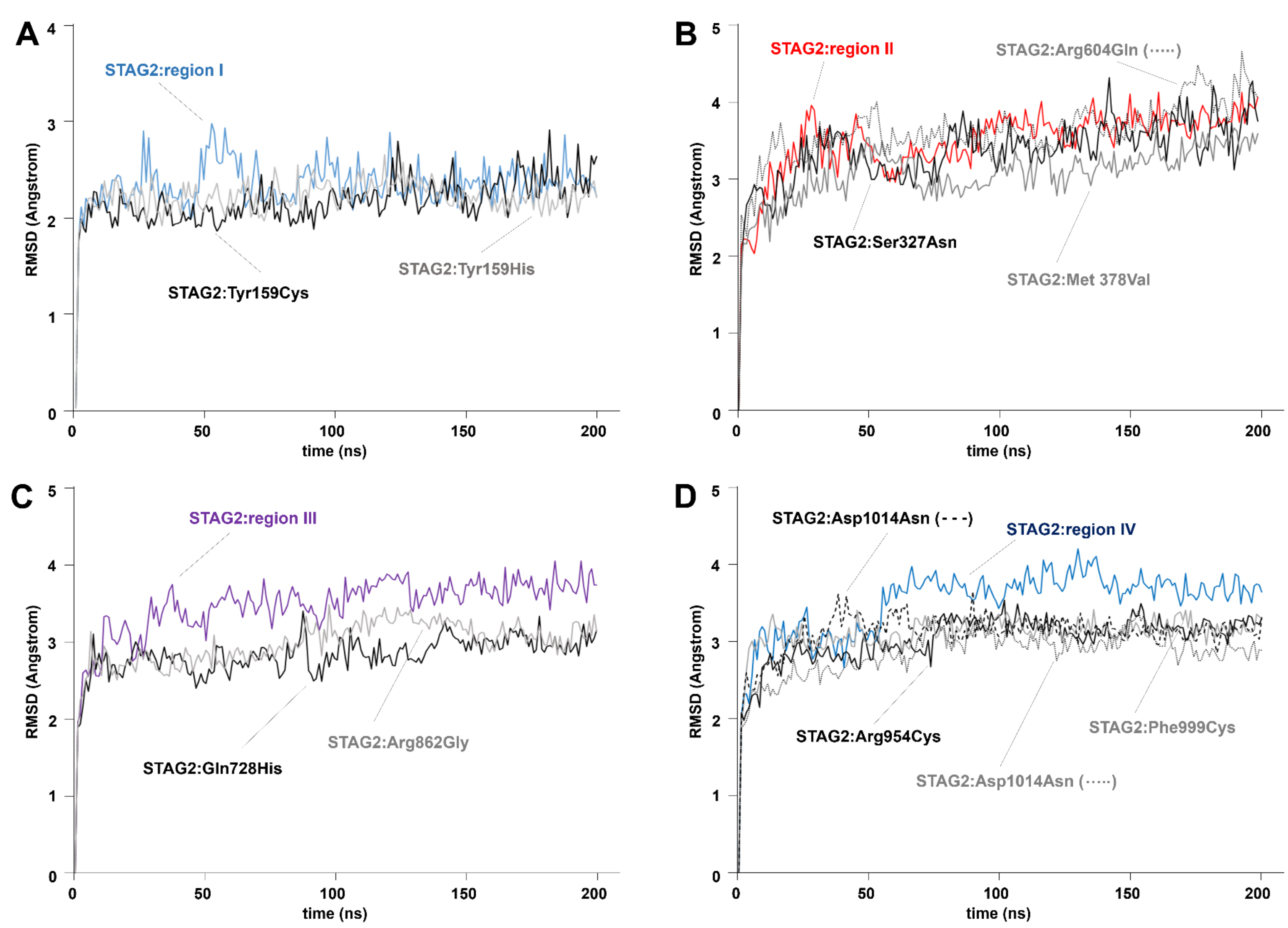

Supplementary Figure S2. Comparison of RMSD values measured over 200 ns of the molecular dynamics trajectory of each of the four regions of STAG2 in the wild-type protein (line colors as in Supplementary Fig. S1) and in the variants located in each of the regions (in different shades of gray and trace types). A. RMSD values of region I of STAG2 wild type and the STAG2:Tyr159Cys and STAG2:Tyr159His variants. B. RMSD values of region II of STAG2 wild-type and the STAG2:Ser327Asn, STAG2:Met378Val and STAG2:Arg604Gln variants. C. RMSD values of region III of STAG2 wild-type and the STAG2:Gln728His and STAG2:Arg862Gly variants. D. RMSD values of region IV of STAG2 wild-type and the STAG2:Arg954Cys, STAG2:Phe999Cys, STAG2:Lys1009Asn and STAG2:Asp1014Asn variants. None of the simulated variants show RMSD values that are significantly higher than those for the wild-type regions, indicating that the amino acid changes analyzed do not generate large variations in the internal structure of the protein.

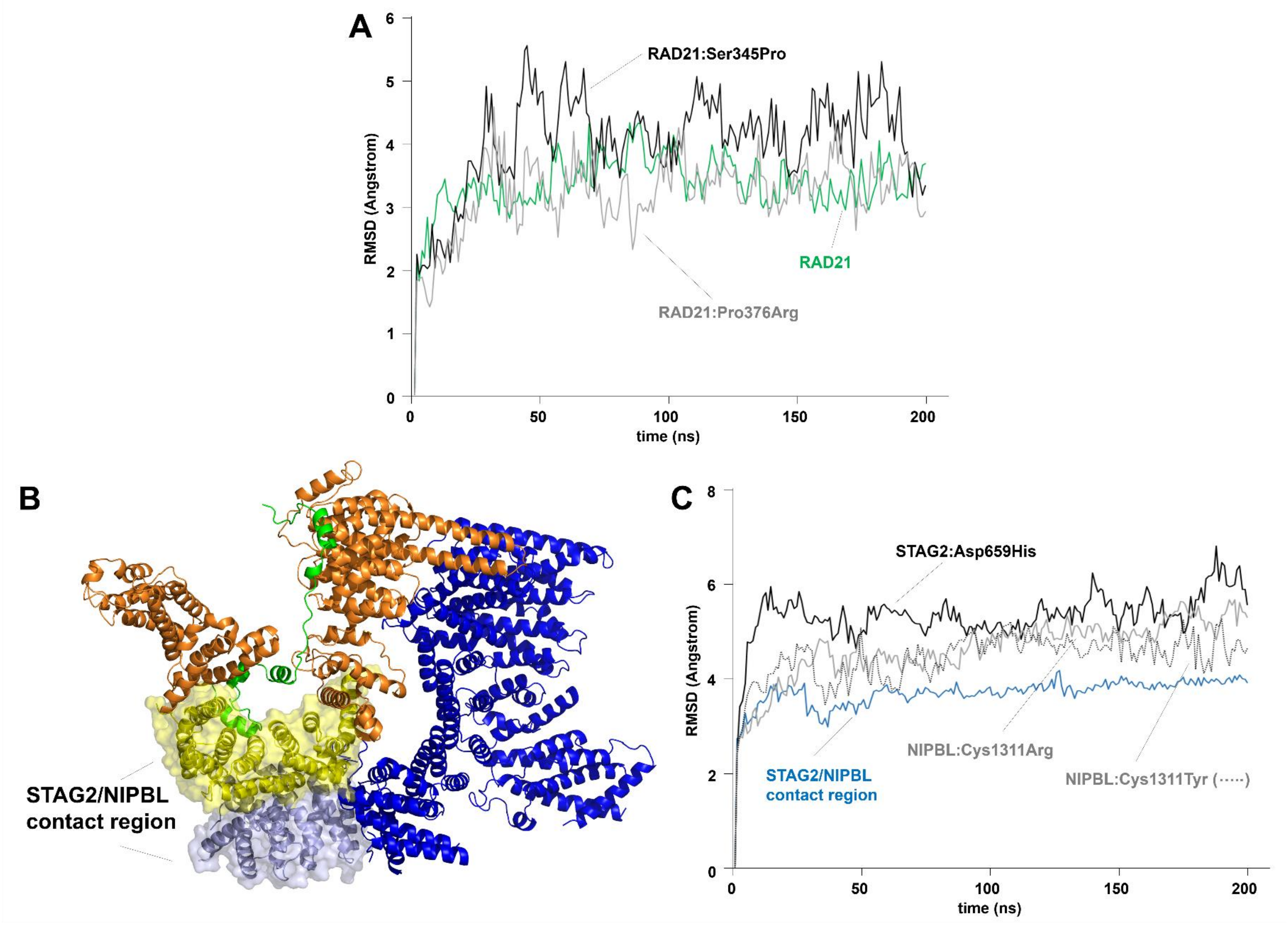

Supplementary Figure S3. A. Comparison of RMSD values measured over 200 ns of the molecular dynamics trajectory of RAD21 (green) and the RAD21:Ser345Pro and RAD21:Pro376Arg variants. The trajectory of the RAD21:Ser345Pro variant shows high RMSD values, indicating a significant change in the structure of the protein segment. B. Model of the interaction between STAG2 (orange) and NIPBL (blue). A region composed of the two contact structures of both proteins (in yellow and cyan) is highlighted. In this region, where the amino acids Asp659 of STAG2 and Cys1311 of NIPBL are located, the RMSD values along the molecular dynamics trajectory have been measured. C. Comparison of RMSD values measured over 200 ns of the molecular dynamics trajectory of the STAG/NIPBL contact region wild-type (blue) and the STAG2:Asp659His, NIPBL:Cys1311Arg and NIPBL:Cys1311His variants. The three variants lead to remarkable structural changes (especially the STAG2:Asp659His variant), resulting in high RMSD values along the trajectory.
